# Novel Virtual Reality App for Training Patients on MRI-guided Radiation Therapy

**DOI:** 10.1016/j.adro.2024.101477

**Published:** 2024-02-26

**Authors:** Brian D. Gonzalez, Sylvia Choo, Joseph J. Janssen, Jeff Hazelton, Kujtim Latifi, Corinne R. Leach, Shannon Bailey, Heather S.L. Jim, Laura B. Oswald, Morgan Woolverton, Martin Murphy, Edward L. Schilowitz, Jessica M. Frakes, Edmondo J. Robinson, Sarah Hoffe

**Affiliations:** a Department of Health Outcomes and Behavior, Moffitt Cancer Center, Tampa, FL; b Morsani College of Medicine, University of South Florida, Tampa, FL; c Ringling College of Art and Design, Sarasota, FL; d XdooR, Venice, FL; e Department of Radiation Oncology, Moffitt Cancer Center, Tampa, FL; f Center for Digital Health, Moffitt Cancer Center, Tampa, FL; g Center for Advanced Medical Learning and Simulation, University of South Florida, Tampa, FL

## Abstract

**Purpose:**

Patients receiving respiratory gated magnetic resonance imaging-guided radiation therapy (MRIgRT) for abdominal targets must hold their breath for ≥25 seconds at a time. Virtual reality (VR) has shown promise for improving patient education and experience for diagnostic MRI scan acquisition. We aimed to develop and pilot-test the first VR app to educate, train, and reduce anxiety and discomfort in patients preparing to receive MRIgRT.

**Methods and Materials:**

A multidisciplinary team iteratively developed a new VR app with patient input. The app begins with minigames to help orient patients to using the VR device and to train patients on breath-holding. Next, app users are introduced to the MRI linear accelerator vault and practice breath-holding during MRIgRT. In this quality improvement project, clinic personnel and MRIgRT-eligible patients with pancreatic cancer tested the VR app for feasibility, acceptability, and potential efficacy for training patients on using breath-holding during MRIgRT.

**Results:**

The new VR app experience was tested by 19 patients and 67 clinic personnel. The experience was completed on average in 18.6 minutes (SD = 5.4) by patients and in 14.9 (SD = 3.5) minutes by clinic personnel. Patients reported the app was “extremely helpful” (58%) or “very helpful” (32%) for learning breath-holding used in MRIgRT and “extremely helpful” (28%) or “very helpful (50%) for reducing anxiety. Patients and clinic personnel also provided qualitative feedback on improving future versions of the VR app.

**Conclusion:**

The VR app was feasible and acceptable for training patients on breath-holding for MRIgRT. Patients eligible for MRIgRT for pancreatic cancer and clinic personnel reported on future improvements to the app to enhance its usability and efficacy.

## Introduction

Magnetic resonance imaging-guided radiation therapy (MRIgRT) is a novel therapy that integrates adaptive therapy and breath-holding to deliver higher biologically effective doses of radiation to tumors and minimize toxicity to healthy tissue.^1^ However, MRI-related discomfort and distress are major barriers to MRIgRT. Up to 15% of patients experience MRI-related claustrophobia,^2^ 14% cannot complete MRI due to severe MRI-related anxiety,^3^ and 29% to 65% report MR-related complaints.^4^^,^^5^

Patients receiving abdominal MRIgRT cannot be easily sedated because they must intermittently hold a deep breath so that gross tumor volume can be targeted. During MRIgRT, patients are shown an image with one circle depicting the static tumor target and another circle depicting their respiratory motion. Patients are asked to take and hold a deep breath so that the circles are superimposed for as long as they feel comfortable. Practice with this procedure can reduce the time to completion, with one study finding 13% faster final MRIgRT treatments than first treatments.^6^ Thus, training for breath-holding may reduce the time needed to complete MRIgRT, increase willingness to undergo MRIgRT, and reduce MRIgRT-related distress.

Mock MRI training^7^ and mobile apps^8^ have shown promise for preparing children for MRI; however, these approaches may have limited impact for 2 reasons. Mock MRI is typically resource-intensive. Smartphone/tablet apps are portable and require fewer resources but are also less immersive. Virtual reality (VR), defined as “computer-generated, interactive, and highly vivid environments” that are multisensory and immersive,^9^ overcomes both limitations. One study compared receiving simulated MRI in VR vs mock MRI. Patients felt that mock simulation was more realistic than VR, but patients in both groups reported similar levels of discomfort and anxiety,^10^ suggesting VR achieves near-realistic experiences of MRI.^11^

Although VR is promising for improving patient education and experience,^12^ we are unaware of any VR apps designed to prepare patients for MRIgRT. We aimed to develop and pilot-test the first VR app with immersive and gamified experiences to educate, train, and reduce anxiety in patients preparing to receive MRIgRT. We hypothesized that the VR app would be acceptable to patients and clinic personnel, feasible to prepare patients for MRIgRT, and efficacious for training patients on deep inspiration breath-holding.

## Methods and Materials

This quality improvement project was deemed exempt from IRB review. A multidisciplinary project team included 2 pancreatic cancer survivors and experts in radiation oncology, VR development, video game graphic design, behavioral medicine, and digital health technology. A design team received feedback from the larger team and met weekly to review progress on the app's features, gamification, user experience, and user interface.

Designers developed the virtual environment and 3-dimensional assets to be included in the environment. Audio files were developed to play ambient sounds, background music, instructions, and responses to user actions. The design team integrated the environment and assets using Unreal Engine 4.26 (Epic Games, Inc.; Cary, NC). The app was deployed on Meta Quest 2 VR headsets (Meta Platforms, Inc.; Menlo Park, CA), tested, and iteratively refined to ensure the app was immersive, realistic, and easy to use. Lastly, the app was deployed for testing with patients and clinic personnel.

### Procedure

**Figure 1:**
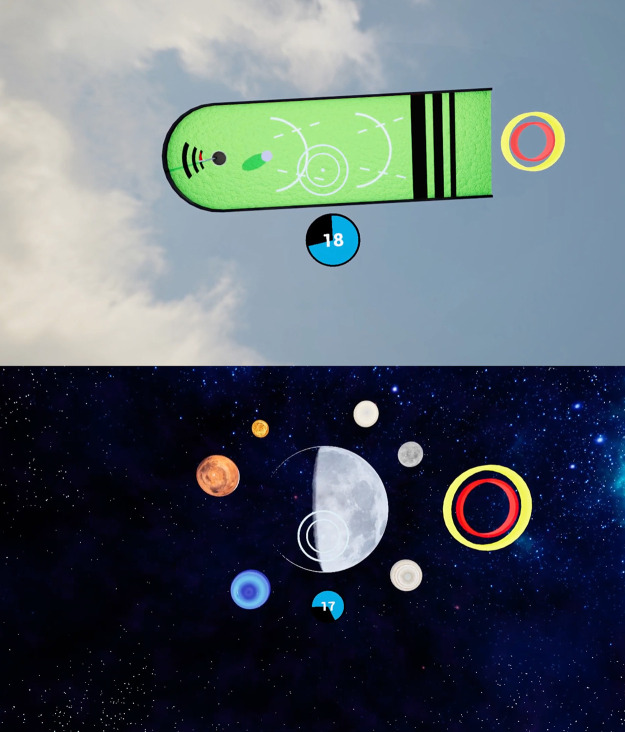
Virtual reality minigames to familiarize users with breath-holding used in MRIgRT. Progress toward the hole and in the moon waxing continue while the user holds their breath to keep the respiratory red circle inside the target yellow circle.

From April through August 2021, we invited clinic personnel and consecutive MRIgRT-eligible patients with pancreatic cancer to use the VR app we developed. Before the simulation, users sat upright on a reclining chair and were familiarized with the app and controls. Users then lay down horizontally before playing 2 minigames (Fig. 1) to promote relaxation and training on the breath-holding technique. Respiratory movement was tracked via a VR controller placed on the user's chest to show how to superimpose the breathing circle over the static target circle using breath-holding. The minigames asked users to hold their breath for 25 seconds to control the distance a golf ball travels on a putting green and to make the moon appear in a night sky.

**Figure 2:**
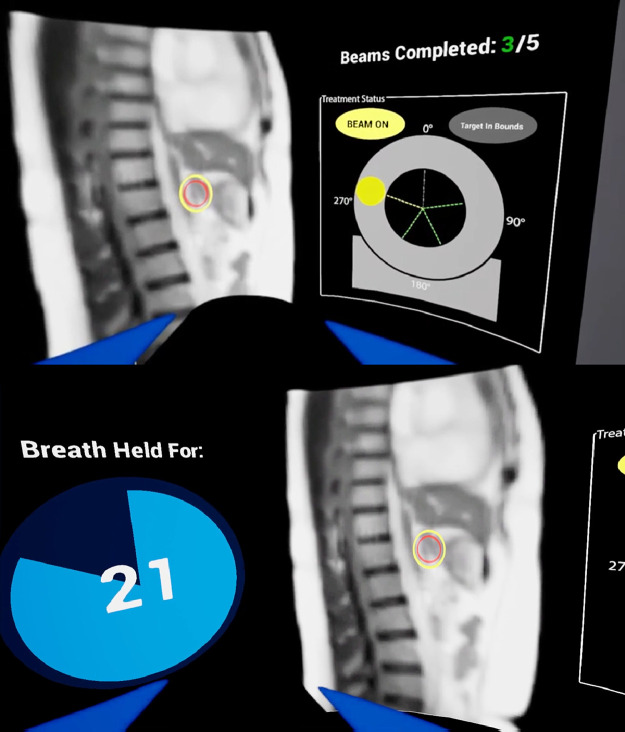
Virtual reality breath-holding training for MRIgRT. These experiences help users practice breath-holding in a simulated MRIgRT environment.

Next, users were introduced to the staff and equipment in an MRI vault. Users were then shown a view from the perspective of a patient lying on an MRI linear accelerator (linac). Figure 2 shows the view while receiving MRIgRT, including breath-holding to localize the tumor in its proper deep breath hold position.

### Measures

Before and after using the VR app, patients were asked to report how anxious they would feel about going through MRIgRT (see supplementary material). After using the app, patients were also asked whether the app helped them understand breath-holding and whether the app helped decrease their anxiety. After using the VR app, clinic personnel were asked whether the app helped them train patients on breath holding.

## Results

### Quantitative results

Of the 22 patients invited to use the VR app, 19 (86%) agreed, including 9 (47%) females and 10 (53%) males. Patients who reported their ages were <50 (n = 3, 17%), 50 to 70 (n = 6, 33%), and 71 to 80 years old (n = 9, 47%). Of the 68 clinic personnel invited to use the VR app, 67 (99%) agreed. Clinic personnel who reported their ages were <30 (n = 12, 19%), 30 to 40 (n = 26, 41%), 41 to 50 (n = 13, 20%), and ≥51 years old (n = 13, 20%). Clinical roles were nurse (n = 3, 4%), physicist (n = 6, 9%), radiation oncologist (n = 3, 4%), radiation therapist (n = 9, 13%), dosimetrist (n = 3, 4%), advanced practice professional (n = 3, 4%), fellow/resident (n = 13, 19%), clinical trial coordinator (n = 5, 7%), and other (n = 22, 33%).

On average, patients completed the app experience in 18.6 minutes (SD = 5.4), and clinic personnel completed the app experience in 14.9 minutes (SD = 3.5). Before starting the VR app, 42% of patients reported they felt at least “somewhat” anxious about MRIgRT. After completing the app, MRIgRT-related anxiety had not changed, with 42% reporting they were at least “somewhat anxious” about MRIgRT. Patients reported the VR app was “extremely helpful” (58%) or “very helpful” (32%) for learning breath-holding for MRIgRT. Of the 18 patients who replied to the question asking how much the VR app helped with decreasing anxiety, 28% reported it was “extremely helpful,” 50% reported it was “very helpful,” and 22% reported it was “somewhat helpful.” Of the 66 clinic personnel who replied to the question asking whether the app would help train patients on breath-holding for MRIgRT, 68% reported it was “extremely helpful,” and 29% reported it was “very helpful.”

### Qualitative feedback

In brief interviews, commonly reported themes were identified related to usability and potential impact. First, many users reported that controllers for tracking users’ breathing sometimes did not detect when the user was holding their breath. Second, users reported that in-app verbal instructions were sometimes unclear or given too quickly, reducing their understanding of the instructions. Third, some users had difficulty finding instructions/interactions that were not centered in the view and others reported difficulty rotating their heads to look at various objects due to comorbidities. Clinic personnel reported the app would likely increase patient understanding of the experience of MRIgRT. Users reported the app would help increase patient self-efficacy in their ability to receive MRIgRT and participate in breath-holding. One patient reported, “I really didn't know what to expect, but this really helps you because it tells me what to do, what to expect.”

## Discussion

Immersive VR technologies can improve the patient experience, increase patient satisfaction, decrease distress, and prepare patients for new procedures. The VR app, designed with input from patients and a multidisciplinary team, used 2 minigames to orient patients to breath-holding before introducing patients to the MRI linac. Users also practiced breath-holding while in the VR MRI linac bore. Most patients felt the prototype decreased their anxiety and taught them to use breath-holding for ≥25 seconds. Similarly, clinic personnel felt the experience would increase preparation and readiness for first-time MRI linac users.

Patients generally reported the app was helpful in reducing anxiety, but MRIgRT-related anxiety did not change from before to after using the VR app. This may reflect a lack of specificity in how the questions were asked. In reporting MRIgRT-related anxiety, patients may have focused on anxiety related to whether MRIgRT would be efficacious rather than on their self-efficacy for completing MRIgRT. Future studies should use validated questionnaires to assess change in specific constructs, such as self-efficacy for completing breath-holding and receiving MRIgRT.

User feedback helped identify VR app improvements. Users reported that the controller was not sufficiently sensitive for breath-tracking. Also, the app's instructions were too rapid or unclear. Lastly, users also reported difficulty moving their head during some minigames. Future versions of the VR app must identify alternate strategies for monitoring respiration, provide slower instructions with visual cues to enhance understanding,^13^ personalize the pace of instructions and feedback to users, and accommodate the needs of patients with limited mobility. The average length of the VR experience was 18.6 minutes, but a shorter experience time may better accommodate busy clinics.

In conclusion, this VR app demonstrated feasibility and acceptability in patients and clinic personnel. Users reported the app was easy to use and helpful for learning breath-holding and preparation for MRIgRT. Users identified opportunities to further enhance the app experience for future development. We anticipate implementing these suggestions in future versions of this app.

## Disclosures

BDG reports fees unrelated to this project from Sure Med Compliance and Elly Health. KL reports fees unrelated to this project from ViewRay. HJ reports fees unrelated to this project from Kite Pharma and SBR Biosciences. JF reports fees unrelated to this project from ViewRay and Boston Scientific. SH reports fees unrelated to this project from VeiwRay.
